# The Relationship Between Perceived Control and Hypothalamic–Pituitary–Adrenal Axis Reactivity to the Trier Social Stress Test in Healthy Young Adults

**DOI:** 10.3389/fpsyg.2021.683914

**Published:** 2021-08-17

**Authors:** Qian Liu, Jianhui Wu, Liang Zhang, Xiaofang Sun, Qing Guan, Zhuxi Yao

**Affiliations:** ^1^Center for Brain Disorder and Cognitive Science, Shenzhen University, Shenzhen, China; ^2^Shenzhen Futian Foreign Languages School, Shenzhen, China; ^3^Shenzhen Institute of Neuroscience, Shenzhen, China; ^4^Key Laboratory of Behavioral Science, Institute of Psychology, Chinese Academy of Sciences, Beijing, China; ^5^Department of Psychology, University of Chinese Academy of Sciences, Beijing, China; ^6^Department of Industrial Engineering, Tsinghua University, Beijing, China

**Keywords:** hypothalamic–pituitary–adrenal, perceived control, Trier Social Stress Test, acute stress, cortisol, uncontrollability

## Abstract

Psychological factors can modulate the hypothalamic–pituitary–adrenal (HPA) axis activity toward stressors. Animal studies demonstrated that uncontrollability was one critical factor associated with HPA axis stress response, but the results in human studies were inconsistent. The current study adopted a standardized laboratory stress induction procedure, the Trier Social Stress Test (the TSST), as the stressor to regulate the objective controllability level, and young adult participants were asked to rate their subjectively perceived control level toward the stressor and measured their cortisol stress responses (*N*=54; 19 females and 35 males) to address this concern. Results showed that participants’ perceived control on the TSST was related to the cortisol stress response. In other words, under the stress of a certain objective controllability level, the lower the subjectively perceived control level, the greater the HPA axis response. This finding suggested that, in addition to objective controllability, subjectively perceived control is a psychological factor that regulates activation of the HPA axis in young adults.

## Introduction

Stress triggers a series of psychophysiological changes in individuals. The hypothalamic–pituitary–adrenal (HPA) axis is one of the most important physiological systems in the human body to cope with acute stressors (Chrousos, 2009). Its final product is cortisol in humans, which can act on multiple parts of the body and the brain, mobilizing energy to handle acute stressors (De Kloet et al., 2005), but may also impair cognitive functions (Lupien et al., 2007).

Controllability is one factor that may influence the HPA axis response to stress (Dickerson and Kemeny, 2004). Many animal studies demonstrated that lack or loss of actual control was associated with HPA axis stress response (e.g., Davis et al., 1977; Dess et al., 1983; Kearton et al., 2020). For example, sheep receiving predictable but uncontrollable aversive stimulus had a higher cortisol compared to the control group (Kearton et al., 2020). In rodents, inescapable shock leads to a prolonged steroid elevation (Davis et al., 1977). And uncontrollable shocks produced significantly greater HPA axis response than controllable shocks in dogs (Dess et al., 1983). Meanwhile, other studies did not find correlation between actual control and HPA axis responses when manipulating control over a stressor in rodents (e.g., Maier et al., 1986; Weinberg et al., 2010).

The results on objective uncontrollability effect in human studies were also inconsistent. On the one hand, some studies found that uncontrollable stress exposure can lead to higher cortisol stress response (Breier, 1989; Croes et al., 1993; Müller, 2011). And a meta-analysis that coded uncontrollability levels of acute psychological stressors in 208 laboratory studies found that motivated performance tasks elicited cortisol responses if they were objectively uncontrollable (Dickerson and Kemeny, 2004). On the other hand, other studies experimentally manipulated objective controllability, but did not find an effect on the HPA axis response (Agrigoroaei et al., 2013; Mayer et al., 2017; Hancock and Bryant, 2018). For example, low objective controllability of a simulated driving task did not lead to a higher cortisol response (Agrigoroaei et al., 2013). And researchers failed to demonstrate an impact of manipulation of objective control on HPA responses in specific phobia participants (Mayer et al., 2017).

One possible reason for this inconsistency may lie in the separation of subjectively perceived control and objective controllability (Weems and Silverman, 2006). While objective controllability refers to whether the subject can change the stressor or the result of behavior, perceived control is the perception of how much individuals’ behaviors can directly influence their life outcomes (e.g., Frazier et al., 2011; Zilioli et al., 2017). The relationship between subjectively perceived control and objective controllability is proposed to be orthogonal (Weems and Silverman, 2006). In facing a stressor of certain objective controllability, individuals’ perceived level of control can vary. However, the above-mentioned studies have mostly focused on objective control level of stressors.

To our knowledge, only a few studies have reported on the relationship between perceived control and cortisol stress response in humans thus far. Bollini et al. (2004) manipulated perceived control in a noise-cognitive stressor and found that perceived control did not affect stress responses. However, this study was conducted in the morning when cortisol levels are declining and as such the normal cortisol circadian rhythm could have obscured the cortisol response due to stress. Mayer et al. (2017) and Müller (2011) reported that lower perceived control was associated with higher salivary cortisol response in the whole sample with the participants either exposed to an objectively controllable or uncontrollable stressor. However, as participants experienced stressors of different objective controllability levels, they may have different subjective levels of stress, which were in turn related to different cortisol response levels, thus undermining the explanation for the relationship between perceived control and cortisol stress response. Such confounding factors can be controlled by using the same stressor of a constant objective control level across participants.

Therefore, in the present study, we aimed to investigate whether perceived control was related to HPA axis response by using the same stressor with a fixed objective controllability level. The Trier Social Stress Test (TSST; Kirschbaum et al., 1993), a standard laboratory-based social psychological stressor involving public speaking and mental arithmetic task, was used. Salivary cortisol concentration across five time points and positive/negative affect of the participants before and after the TSST were taken to measure stress responses. Perceived control was assessed immediately after each participant completed the TSST. Based on previous results (Dickerson and Kemeny, 2004; Müller, 2011), we hypothesized that perceived control would be negatively associated with the cortisol stress response. That is, the higher the perceived control, the smaller the cortisol stress response.

## Materials and Methods

### Participants

A total of 54 healthy students (35 males and 19 females, mean age ± *SE* = 22.57 ± 0.23, mean educated years ± *SE* = 15.89 ± 0.18, and mean body mass index (BMI) ± *SE* = 22.02 ± 0.41) from several universities in Beijing were recruited as participants for the present study. All participants were Chinese. A prescreening process was conducted with the following exclusion criteria: (1) history of psychiatric or neurological disorders; (2) chronic use of any psychiatric, neurological, or endocrine medicine that would affect the central nervous system; (3) any chronic severe mental illness; (4) history of severe head injuries, neurological disease, or endocrine disorder; (5) an irregular circadian rhythm (e.g., sleep during daytime and work at night); or (6) pregnancy. Furthermore, as a previous study showed that alcohol and nicotine affect stress response (Munro et al., 2005), people who excessively drank (more than two alcoholic beverages a day) or smoked (more than five cigarettes a day, referred to von Dawans et al., 2011) were excluded. Participants’ ovulation period was avoided during the experiment as the female physiological menstrual cycle might have a sizeable effect on cortisol secretion (Kajantie and Phillips, 2006). The participants did not take medicines and slept normally in the 24h prior to the experiment. The analysis of the present study was based on a secondary analysis of data from “a project on psychophysiological responses to acute stress” (Xin et al., 2017). All participants provided written informed consent and were paid for their participation. This experiment was approved by the Ethics Committee of Human Experimentation in the Institute of Psychology, Chinese Academy of Sciences.

### General Procedure

**Figure 1:**
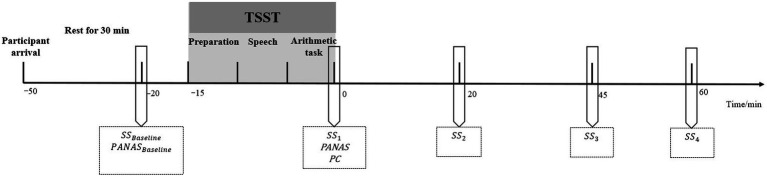
General procedure. TSST, The Trier Social Stress Test; SS, saliva sampling; PANAS, the Positive and Negative Affect Scale; and PC, perceived control assessment. 0 indicates the end of the TSST.

The experiment was conducted in the afternoon from 1:00 to 5:00pm to avoid the effect of circadian rhythms on cortisol levels (Van Cauter et al., 1996; Kudielka et al., 2004). The participants were asked not to perform any strenuous exercise 1h before arriving at the laboratory or to consume anything except for water. The participants reported that they strictly followed these experimental requirements. Upon arrival, they rested in a quiet room for 30min. After the rest period, their saliva samples were collected, and the positive and negative affect state was measured using the Positive and Negative Affect Scale (PANAS) for baseline. Then, the participants performed a 15-min TSST task. After completing the TSST, they returned to the resting room. The visual analog scale for perceived control (PC) and the PANAS were immediately completed. Further, as previous studies have reported that the peak salivary cortisol levels would appear around 10–20min after cessation of the TSST (Kirschbaum et al., 1993; Langer et al., 2020; Keenan et al., 2021; for a review, see Kudielka et al., 2007), saliva samples were collected four times at 0, 20, 45, and 60min after the TSST as referred to measurement settings of previous studies using the TSST as the acute stressor (Johnson et al., 2019; Schmalbach et al., 2020; Figure 1).

### Stress Induction

The TSST was first proposed by researchers at the University of Trier and has been a classic paradigm used to induce acute stress in a laboratory (Kirschbaum et al., 1992, 1993). The modified version of the TSST used in the present study can induce stress response similar to that of the original (Buchanan et al., 2012). Furthermore, the TSST is considered a mature and practical psychological stress test as it provides a stressful environment with uncontrollable and socially threatening aspects (Dickerson and Kemeny, 2004; Rosenbaum et al., 2018). The modified version of the TSST consists of three 5-min phases: preparation, speech, and mathematical mental arithmetic phases. In the preparation stage, the participants were asked to prepare a 5-min speech in which they should defend themselves against charges of stealing made by supermarket managers and security guards, which is different from the original TSST where participants were instructed to prepare a speech for a mock job interview to convince the group that they are ideal candidates for a job (Kirschbaum et al., 1993). Participants were allowed to prepare and take down notes using a pen and paper, but they were not allowed to bring the materials outside the resting room. After the preparation, they were escorted to the speech room, where three experimental assistants (two females and one male) wearing white coats were waiting. The experimenters pretended to be supermarket managers and communicated with the participants neutrally with no language or physical feedback. After the speech, the participants performed the mental arithmetic task wherein they were required to continuously subtract 13 from the number 1022 as quickly and accurately as possible. Once they made a mistake, they had to recalculate from 1022. Throughout the TSST task, they stood in front of the managers, spoke into a microphone, and were recorded by a video camera.

### Measurements

#### Physiological Indicator

Cortisol is a physiological indicator of stress response. Saliva samples were collected using Salivettes, an international disposable saliva collection tube (Sarstedt, Rommelsdorf, Germany), and frozen at −22°C until analysis. The saliva samples were thawed and centrifuged at 3000rpm for 10min before analysis. Salivary cortisol levels were measured with electrochemiluminescence immunoassay (Cobas e 601, Roche Diagnostics, Numbrecht, Germany). The lower sensitivity of cortisol detection was 0.500nmol/l, the test range was 0.5–1750nmol/l, and both inter- and intra-assay variations were less than 10%. Owing to insufficient saliva, three values were missing. The missing values were replaced through estimation based on the group means and standard deviation of the cortisol sample at the time and the mean of the available cortisol samples of the respective participants (Booij et al., 2013).

#### Positive and Negative Affect Scale

The PANAS (Watson and Clark, 1988) was used to measure participants’ positive and negative affective state. The scale consists of 20 items, with 10 depicting positive affects (interested, excited, and enthusiastic) and 10 depicting negative affects (distressed, nervous, and scared). Ratings were based on a 5-point Likert scale from 1 (not at all) to 5 (very much). The participants rated each item according to the current affective status at the baseline and at the end of TSST. The positive and negative state’s scores were computed separately, with the overall sum scores of each ranging from 10 to 50. The higher the score, the stronger the emotion. The scale had satisfactory internal consistency reliability (Cronbach’s Alpha was 0.88 and 0.87 for PA and NA, respectively), as reported by Watson and Clark (1988).

#### Perceived Control

The participants completed a visual analog scale of perceived control immediately after the cessation of the TSST task. The scale contains two questions: one assessing the sense of control over the speaking task and the other assessing the sense of control over the mental arithmetic tasks during the TSST. Participants were asked “How much control do you feel you had during the public speaking task/the mental arithmetic task?” They were required to mark a level of control over a line measuring 0–100, with 0 indicating completely uncontrollable and 100 denoting completely controllable. The larger the value, the higher the sense of control. The average control score of speech and mental arithmetic task were considered the subjectively perceived control of the participant.

### Data Analysis

To test whether the TSST successfully induced physiological stress response, one-way repeated-measures ANOVA was first conducted for salivary cortisol with time point being within-subject variable (five time points for salivary cortisol; refer to Figure 1 and “General Procedure”). The Greenhouse–Geisser correction was used if the sphericity assumption was violated. Partial *ƞ*^2^ was included to measure the effect size. Post-hoc multiple comparisons were conducted using the Bonferroni adjustment. Area under the curve with respect to increase (AUCi) of salivary cortisol was computed following the trapezoid formula to serve as the index of the HPA axis stress response (Pruessner et al., 2003).

To test whether the TSST successfully induced emotional response to stress, a paired sample t-test was first performed on the positive and negative emotional state scores between the baseline and the end of the TSST. Then, positive and negative affect change (ΔPA, ΔNA) was calculated by subtracting the baseline from the score measured at 0min at the end of TSST.

Hierarchical regression analyses were used to test the relationship between perceived control and salivary cortisol response. In the regression model, salivary cortisol AUCi was the dependent variable. Perceived control was entered in Step 1. As sex has been reported to influence stress responses (Kudielka and Kirschbaum, 2005), sex was entered as control variable in Step 2. For BMI which has been found to relate to stress responses (e.g., Jones et al., 2012; Miller et al., 2013), BMI was entered as control variable in Step 3. Furthermore, it is possible that it is not perceived control that relate to heightened cortisol responses, but rather that participants felt more stressful in general and therefore felt more negative feelings, which are in turn related to increased cortisol responses. Thus, ΔNA was entered as control variable in Step 4 to test whether the association between perceived control and cortisol stress response was just because that participants felt more distressing toward the TSST. As we were focusing on individual differences in cortisol responses to stress, we included all participants in data analysis, although four out of 54 participants did not show increased salivary cortisol levels after the TSST, which was consistent with previous studies using the TSST as a stressor (Kirschbaum et al., 1993; Schommer et al., 2003; van den Bos et al., 2009; Wu et al., 2017; Lin et al., 2020; for a review, see Kudielka et al., 2007). Statistical analysis was performed by SPSS 24 software. The significance level was 0.05, and all reported results were from two-tailed tests.

## Results

### Descriptive Results

**Figure 2:**
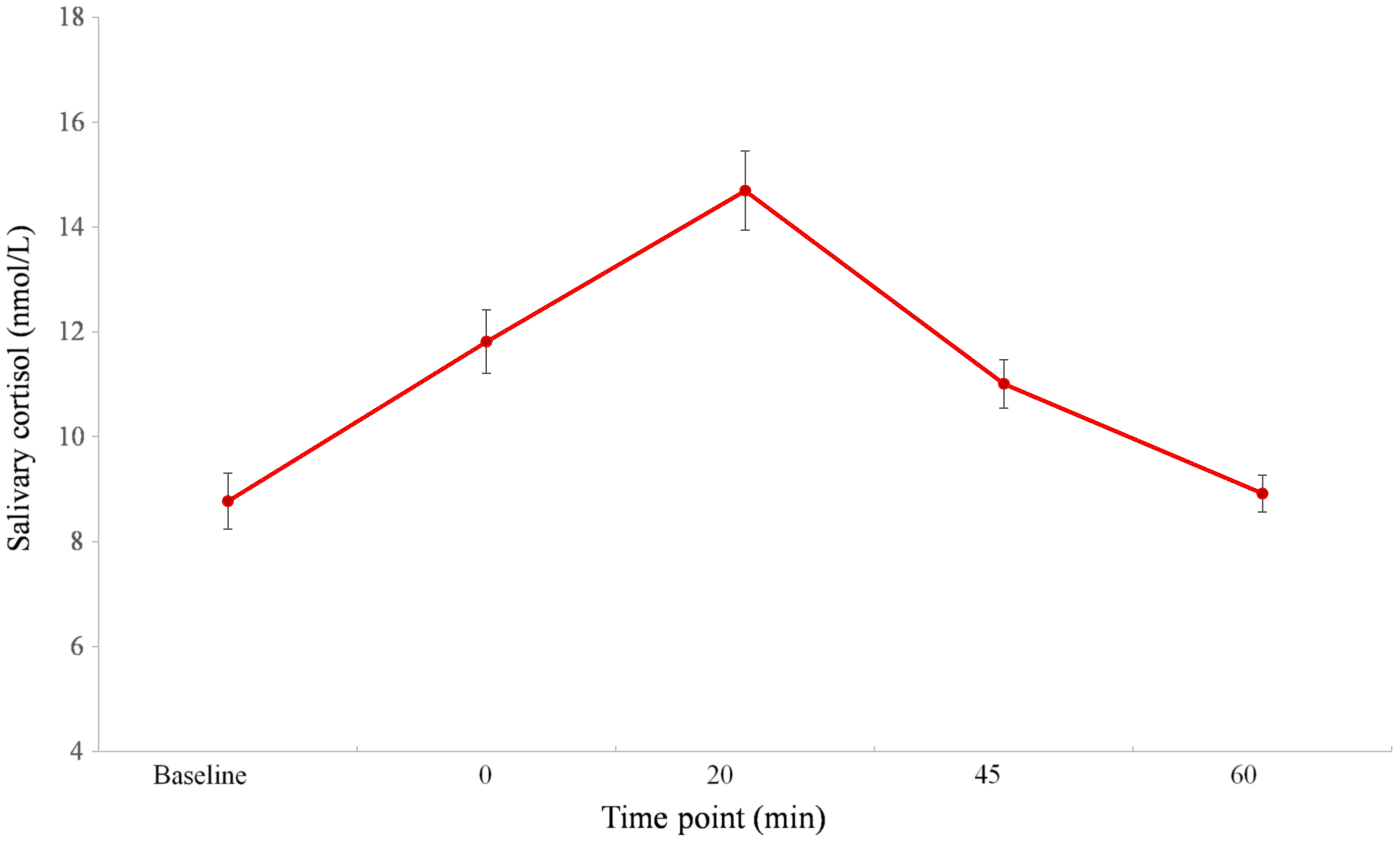
Cortisol response before, during, and after TSST (adapted from Xin et al., 2017). Error bars show the standard error of the mean. The number 0 indicates the end of the TSST.

The mean with SE of salivary cortisol levels is shown in Figure 2 (adapted from Xin et al., 2017). The main effect of time point for salivary cortisol was significant, *F* (4, 212)=40.465, *p*<0.001, partial *ƞ*^2^=0.433. Post-hoc analysis indicated that the maximum salivary cortisol response occurred 20min after the TSST, which was higher than any other period (*ps*<0.001). At time point 0 (*i.e*., immediately after the TSST), the salivary cortisol level was higher as compared to the baseline (*p*<0.001). At 45min after the TSST, cortisol began to decrease, but it was higher compared to the baseline (*p*<0.001). No significant difference was found between the baseline and time point of 60min after the TSST (*p*>0.05). Moreover, the mean value (± *SE*) of the cortisol AUCi was 4.54 (± 0.77).

For the emotion response to stress, the negative emotional level after the TSST (*M*=16.72±0.76) was significantly higher than the baseline (*M*=13.44±0.60), *t* (53)=−4.84, *p*<0.01. However, there was no significant difference between positive emotions at the baseline (*M*=27.56±0.83), and after the TSST (*M*=26.70±1.11), *t* (53)=1.2, *p*>0.05. The mean value (± *SE*) of ΔNA was 3.28 (± 0.68), and the range was from −6 to 26. For the perceived control of the TSST, the mean value (± *SE*) was 53.00 (± 0.23) and the range was 19 to100. Perceived control and cortisol at baseline were correlated (*r*=0.34, *p*<0.05).

### Regression Results

**Figure 3:**
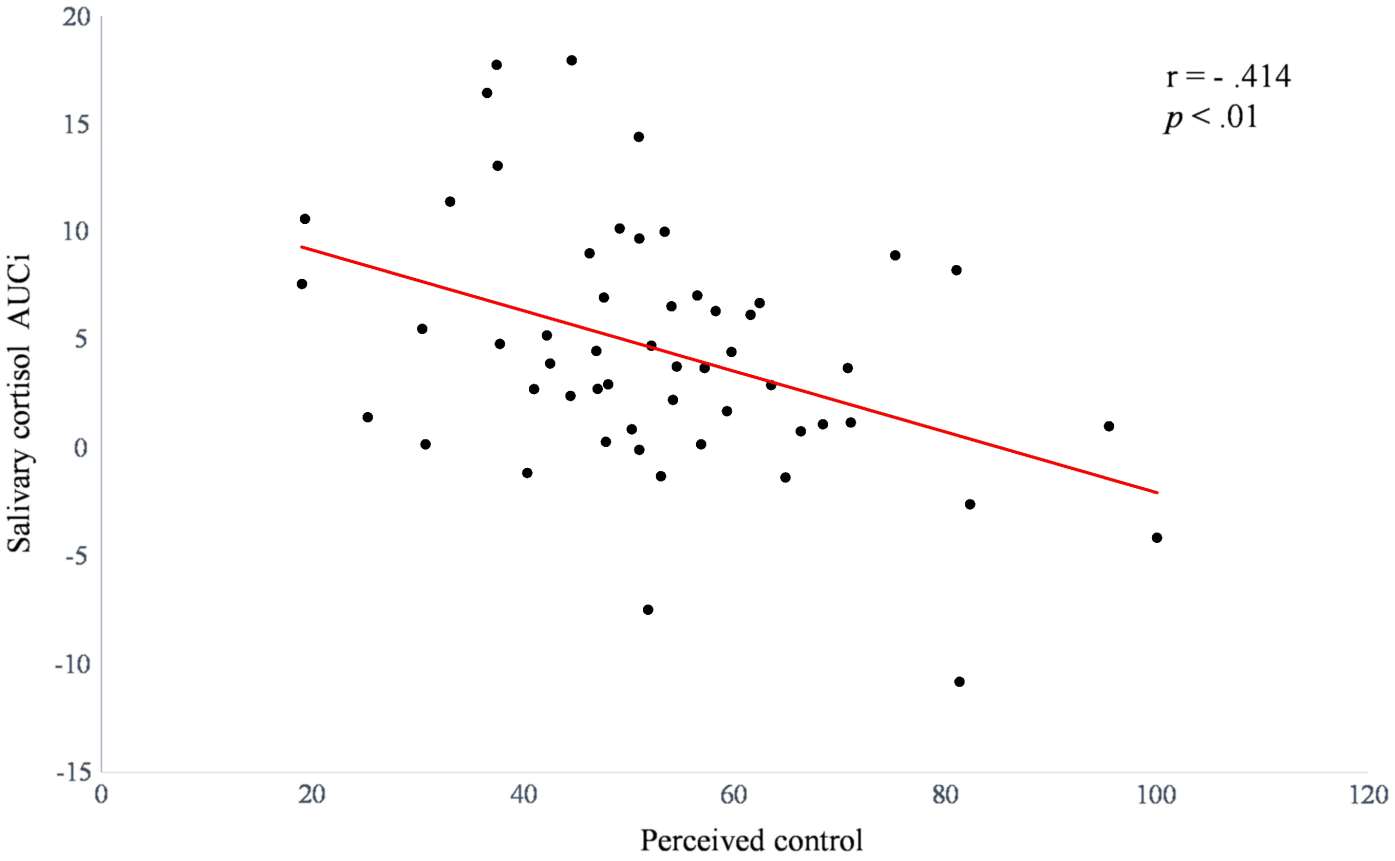
Scatter plot of the bivariate correlation between perceived control and salivary cortisol AUCi to acute psychosocial stress (*n*=54).

Regression analysis results for the cortisol response to stress are presented in Table 1. The result showed that higher perceived control predicted lower cortisol response without covariates (*β*=−0.414, *R*^2^=0.171, *t*=−3.277, *p*=0.002). After controlling for covariates (*i.e*., sex, BMI, and ΔNA), perceived control significantly added 3.7% of the explained variance (*β*=−0.424, *R*^2^=0.208, *t*=−3.093, *p*=0.003), which demonstrated that perceived control was a prime predictor of the cortisol response. To illustrate the relationship between perceived control and the salivary cortisol response to stress, we presented scatter plot of the bivariate correlation between perceived control and salivary cortisol AUCi to the TSST (*r*=−0.414, *p*<0.01; Figure 3).

**Table 1:** Hierarchical regression analyses on HPA axis response to the TSST.

	Predictors	*β*	*t*	*p*	*R* ^2^	Δ*R*^2^	*F*	sig-*F*
Model 1	Perceived control	−0.414	−3.277	0.002	0.171	0.171	10.738	0.002
Model 2	Perceived control	−0.444	−3.51	0.001	0.205	0.034	6.572	0.003
	Sex	−0.186	−1.471	0.147				
Model 3	Perceived control	−0.444	−3.474	0.001	0.205	0.000	4.296	0.009
	Sex	−0.190	−1.281	0.206				
	BMI	−0.007	−0.05	0.960				
Model 4	Perceived control	−0.424	−3.093	0.003	0.208	0.003	3.218	0.020
	Sex	−0.189	−1.261	0.213				
	BMI	−0.024	−0.156	0.877				
	∆NA	0.061	0.439	0.663				

*The outcome measure was the cortisol AUCi during the TSST. Perceived control was included in Step 1. Sex was then included in Step 2. Body mass index (BMI) was further included in Step 3. Change in negative affect (ΔNA) was finally included in Step 4*.

## Discussion

The current study investigated the relationship between subjectively perceived control and the HPA axis activation to acute stress in healthy young participants using the TSST as a stressor. The results showed that salivary cortisol levels and negative affect increased substantially due to the TSST task. Most importantly, the levels of cortisol stress responses were negatively related to the individuals’ perceived control on the stressor. That is, the higher the perceived control on the stressor, the smaller the salivary cortisol AUCi in response to the TSST. Furthermore, the association between perceived control levels and cortisol stress responses remained significant after controlling for sex, BMI, and a general stress level indexed by changes in negative affect.

The main finding of the current study was that the higher perceived control over the stressor one had, the lower salivary cortisol level one would exhibit in facing with an acute stressor. The result indicated that subjective uncontrollability was associated with stress-induced HPA axis activation. Many animal studies have found that objective uncontrollability could affect neurobiology after stress, manifested as elevated cortisol levels (e.g., Davis et al., 1977; Dess et al., 1983; Kearton et al., 2020). However, other studies found that control is not correlated with HPA axis responses when manipulating actual control over a stressor in rodents (e.g., Maier et al., 1986; Weinberg et al., 2010). Actual control may be different with perceived control, which, however, is difficult to measure in rodents. Here, we demonstrated a relationship between subjective control and HPA axis response to stress in humans.

The current finding can provide explanation for the inconsistency in human studies on the relationship between controllability and HPA stress response. Previous studies manipulated the objective control level of stressors and yielded inconsistent results in terms of HPA axis stress responses. While some studies found main effect of objective uncontrollability on cortisol stress response (Breier, 1989; Croes et al., 1993; Müller, 2011), other failed to demonstrate a significant influence on the HPA axis response (Agrigoroaei et al., 2013; Mayer et al., 2017; Hancock and Bryant, 2018). We supposed that this inconsistency maybe caused by different subjective control level of the participants. That is, subjective controllability is an important independent factor for HPA axis response to stress besides objective controllability. However, the few previous studies measuring perceived control levels had used stressors of different objective control levels (Müller, 2011; Mayer et al., 2017), which can be a confounding factor undermining the relationship between perceived control and HPA axis response to stress. In the current study, we exposed all participants to the same stressor, thus controlling for the objective control level, and demonstrated a negative association between subjective control and cortisol stress response.

The negative association between perceived control and cortisol stress response has two potential explanations. First, perceived control may have reflected one’s control beliefs and self-efficacy. Control belief refers to a general belief about control over desired outcomes (Lachman and Agrigoroaei, 2012). Low preexisting control belief is generally associated with high cortisol response, but it may induce the highest cortisol response when the actual situation conflicts with the situational expectations for control (Agrigoroaei et al., 2013). Meanwhile, self-efficacy is individuals’ faith over their capability to generate and regulate events in their lives (Bandura, 1982). Individuals with stronger self-efficacy generally believed that they have more control over their lives and tend to report more controllability over current stressors. However, individuals with low control beliefs thought that environmental events were caused by fate, luck, or opportunity (Bollini et al., 2004). They tend to lose control when experiencing stress and are more likely to show vulnerability over stressors, which may explain the implicit relationship between the perceived control and depression susceptibility (Lachman and Weaver, 1998). The perceived control on the public speech and public mental arithmetic task in the current study may have reflected the inherent level of one’s control beliefs and self-efficacy. Individuals with low perceived control were more likely to exaggerate the threat of the stressor and, therefore, more likely to have an overreaction of cortisol to stress.

Second, the correlation between perceived control and cortisol response may be based on common neurobiological mechanisms. Both animal and human studies found that perceived uncontrollability was related to the function of ventromedial prefrontal cortex (vmPFC) region, which was also critical to HPA axis activation to stress (Admon et al., 2013). For example, the presence of control could activate the area of the vmPFC (Machida et al., 2018). A phobic exposure study found that people exhibited higher vmPFC activity when they had control over the presentation of horror videos than those without control (Kerr et al., 2012). Meanwhile, the PFC played a crucial regulatory role in HPA axis stress response (Kern et al., 2008). Activated vmPFC could inhibit HPA axis stress responses in relevant cortical regions, such as the amygdala and the dorsal sulcus (Amat et al., 2005), whereas low PFC activation was typically associated with higher cortisol levels (Kaldewaij et al., 2019). Thus, the relationship between perceived control and cortisol response might be explained by those individuals with more perceived control over the TSST task having a greater activation of vmPFC, which consequently inhibited the stress response of the HPA axis.

Despite these implications, this study has some limitations. First, the generalizability of our findings was limited by solely investigating only young Chinese undergraduates. The relationship between subjectively perceived control and HPA axis response should be examined in a broader demographic sample. Second, although a significant correlation exists between the visual analog self-reported measure of perceived control and the stress responses, more effective and objective cognitive neural indicators representing perceived control are required. Third, the sample size of this study is relatively small and we only measured salivary cortisol to reflect the HPA axis stress response. Future studies should make a broader biological measurement of the HPA axis (e.g., plasma cortisol) with a bigger sample size to test the reliability of subjective control and the HPA axis stress response relationship. Forth, we measured perceived control after the cessation of stress exposure. The perceived control measurement setting effect should be investigated in future study as previous study have found that the time setting of subjective measurement in stress study can influence the results of concern (e.g., Hellhammer and Schubert, 2012). Fifth, we controlled change in negative affect to test whether the association between perceived control and cortisol stress response was just because that participants felt more distressing toward the TSST. Future studies can take a more direct measurement of subjective stressfulness/distressing as a control factor.

In summary, this study found that, under the stressors of the same objective control level, the young participants with high perceived control tend to have lower levels of salivary cortisol response to the stressor. The association between perceived control and salivary cortisol level remained significant after controlling for sex and negative emotional response due to the TSST tasks. This result could explain the inconsistencies within the relationship between uncontrollability and HPA activation in human studies while highlighting the importance of subjectively perceived control in stress response in young people. Future research should focus on design interventions to enhance individual perceived control of stressors and examine the effectiveness of these interventions.

## Data Availability Statement

The original contributions presented in the study are included in the article/supplementary material, further inquiries can be directed to the corresponding author.

## Ethics Statement

The studies involving human participants were reviewed and approved by the Ethics Committee of Human Experimentation in the Institute of Psychology, Chinese Academy of Sciences. The patients/participants provided their written informed consent to participate in this study.

## Author Contributions

QL analyzed the data and drafted the manuscript. JW participated in the design of study and the interpretation of the data. XS collected the data. QG participated in the interpretation of the data. ZY designed the study, collected the data, and revised the manuscript. All authors contributed to the article and approved the submitted version.

## Conflict of Interest

The authors declare that the research was conducted in the absence of any commercial or financial relationships that could be construed as a potential conflict of interest.

## Publisher’s Note

All claims expressed in this article are solely those of the authors and do not necessarily represent those of their affiliated organizations, or those of the publisher, the editors and the reviewers. Any product that may be evaluated in this article, or claim that may be made by its manufacturer, is not guaranteed or endorsed by the publisher.
